# Dose-Dependent and Species-Specific Effects of Wood Distillate Addition on the Germination Performance of Threatened Arable Plants

**DOI:** 10.3390/plants12173028

**Published:** 2023-08-23

**Authors:** Riccardo Fedeli, Tiberio Fiaschi, Claudia Angiolini, Simona Maccherini, Stefano Loppi, Emanuele Fanfarillo

**Affiliations:** 1 Department of Life Sciences, University of Siena, 53100 Siena, Italy; riccardo.fedeli@student.unisi.it (R.F.); angiolini@unisi.it (C.A.); maccherini@unisi.it (S.M.); emanuele.fanfarillo@unisi.it (E.F.); 2 NBFC, National Biodiversity Future Center, 90100 Palermo, Italy; 3 BAT Center—Interuniversity Center for Studies on Bioinspired Agro-Environmental Technology, University of Naples ‘Federico II’, 80138 Napoli, Italy

**Keywords:** arable weed, biodiversity, plant conservation, pyroligneous acid, segetal plant, sustainable agriculture, wood vinegar

## Abstract

Wood distillate (WD) is a bio-based product applied to crop plants for its known action in terms of growth promotion and yield enhancement, but studies are lacking on its effects on the germination of arable plants. To test such effects, we applied WD at six different concentrations on the diaspores of three threatened arable plants: *Bromus secalinus*, *Centaurea cyanus*, and *Legousia speculum-veneris*. For all the studied species, the effect of WD was dose-dependent and species-specific. In *B. secalinus*, the germination percentage (GP) decreased at 0.125% WD but then remained stable at higher concentrations up to 1%. At 2% WD, almost no germination was observed. Mean germination time (MGT) was not influenced up to 1% WD but significantly increased at 2% WD. The germination rate index (GRI) and germination energy (GE) remained unaffected up to 1% WD but decreased at 2% WD. In *C. cyanus*, WD had no effects on GP and GE at any concentration. MGT showed no difference with the control up to 1% WD, but significantly increased at 2% WD. GRI increased only at low concentrations (0.125% and 0.25%). The germination performance of *L. speculum-veneris* was unaffected up to 0.25% WD for all the tested parameters. From 0.5% WD, a reduction in GP, GRI, and GE and an increase in MGT were observed. At 2% WD, germination was totally blocked. Our results suggest that using WD at low concentrations (<0.5%), those commonly used in arable crops, does not affect the germination of the three investigated plant species.

## 1. Introduction

Arable plants are those plant species that colonize arable land, living among crops. They are usually annual species adapted to the regular disturbance of arable fields, and their life cycle is synchronized with that of the crop they live with [1]. Although farmers usually consider them undesired weeds, their value for biodiversity and ecosystem services is now widely recognized [2,3,4]. The presence of species-rich but well-balanced arable plant communities, i.e., with a low abundance of each species, can significantly reduce yield losses compared to species-poor communities dominated by few very competitive and harmful species [5]. Thus, the latest direction in sustainable weed management points towards finding neutral arable plant communities, i.e., those coexisting with crops without negatively affecting crop yield and quality compared to weed-free conditions, more than at detecting solutions for the eradication of spontaneous vegetation in arable fields [6].

With the spread of modern, intensive agricultural practices and the gradual disappearance of traditional farming, a large number of formerly common arable plants have undergone a noticeable regression in many areas [7,8,9]. Moreover, arable plant communities were subjected to a major decrease in species richness and to relevant shifts in species composition, with an increase in frequency and abundance of competitive, nitrophilous, and herbicide-tolerant taxa [10,11,12]. For these reasons, several arable plant species and one arable habitat are currently red-listed in Europe [13,14]. Such a decline in arable plant diversity was especially due to the introduction of herbicides and chemical fertilizers, which underlines the need to find biodiversity-friendly products in agriculture [7]. It is well established that the application of chemical fertilizers to enhance crop yield negatively affects the growth of arable plants, particularly during their early developmental stages [15,16]. This detrimental effect poses a threat to the biodiversity of arable lands, potentially impacting pollinating insects and delaying or even inhibiting the flowering and development of crop species [17]. Therefore, there is an urgent need to explore alternative approaches that improve crop performances while not damaging the spontaneous plant diversity of arable fields, in line with the targets of the Agenda 2030 to achieve a sustainable food production and to develop resilient agricultural practices [18].

Wood distillate (WD) is currently a very promising sustainable biostimulant, whose use is allowed in organic farming [19]. It is obtained from the condensation of vapors produced during the pyrolysis of woody biomass for green energy production. Several studies have proved that WD exerts a significant influence on cultivated plants, leading to increased yields and improved quality of their edible parts [20,21,22]. There is also evidence that WD can largely mitigate the harmful effects of high ozone concentrations on crop plants [23]. However, the knowledge of the effects of WD on spontaneous plant diversity is still limited to their seedling emergence and first-stage growth under laboratory conditions [24].

The scientific literature on the effects of WD on germination mainly deals with cultivated plants. Studies have highlighted the absence of negative effects of WD at low concentrations on the germination performance of rice (*Orzya sativa* L.) and kura clover (*Trifolium ambiguum* M. Bieb) [25,26]. Conversely, there is evidence that low-dose WD enhances the germination performance of cucumber (*Cucumis sativus* L.), lettuce (*Lactuca sativa* L.) [27], chickpea (*Cicer arietinum* L.), and basil (*Ocimum basilicum* L.) [28]. The mechanism of action of WD on germination is primarily linked to its content in several bioactive compounds such as butanolide, a molecule belonging to the karrikin family of phytohormones [29,30], which has a positive effect [31]. This compound likely plays a crucial role in promoting seed germination, since karrikin phytohormones are known to stimulate germination and to regulate seedling photomorphogenesis [32,33]. With regard to rare and threatened arable plants, low-dose WD has been found to not have negative effects on seedling emergence and first-stage growth in *Bromus secalinus* L., *Centaurea cyanus* L., *Lathyrus aphaca* L., *Legousia speculum-veneris* (L.) Chaix, and *Scandix pecten-veneris* L. [24].

Treating crops with WD implies a possible indirect influence of this product on the plants spontaneously growing in arable fields. However, to the best of our knowledge, no study has so far investigated the effects of WD on the germination performance of arable plants. Thus, in this study we tested whether WD at different concentrations affects the germination parameters of three arable plant species of European conservation interest [7], namely *Bromus secalinus*, *Centaurea cyanus*, and *Legousia speculum-veneris*. We tested its effects at the concentrations used to enhance crop growth and yield (0.25% and 0.5%), at lower concentrations (0.125%), and at the concentrations used to remove weeds (1% and 2%).

## 2. Results

We observed that different concentrations of WD had different and species-specific effects on the investigated germination parameters (Table 1).

**Figure 1:**
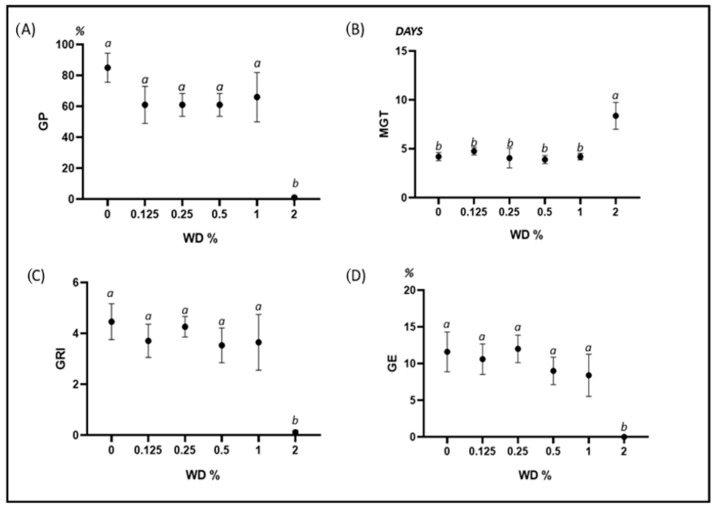
Error bar charts (mean ± standard deviation) showing the differences in the investigated germination parameters under different WD treatments on *Bromus secalinus*. (**A**) GP: germination percentage; (**B**) MGT: mean germination time; (**C**) GRI: germination rate index; (**D**) GE: germination energy; WD: wood distillate. Different letters indicate statistically significant differences at *p* < 0.05.

In B. secalinus, we observed a reduction of GP at 0.125% WD with respect to the control. GP remained stable at increasing concentrations, up to 1% WD. At 2% WD, the germination was almost completely inhibited. MGT was not affected by the treatments up to 1% WD, while it significantly increased at 2% WD. GRI and GE showed a very similar trend, with no effects observed up to 1% WD, while at 2% WD, both parameters approached zero (Figure 1).

**Figure 2:**
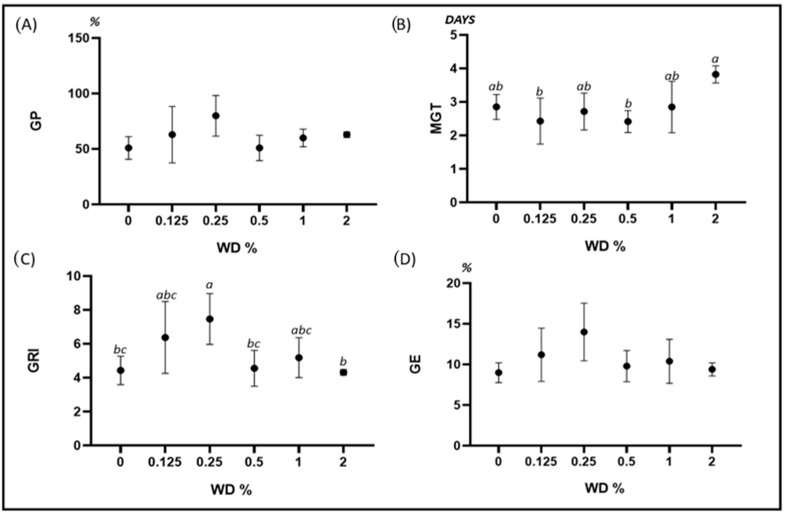
Error bar charts (mean ± standard deviation) showing the differences in the investigated germination parameters under different WD treatments on *Centaurea cyanus*. (**A**) GP: germination percentage; (**B**) MGT: mean germination time; (**C**) GRI: germination rate index; (**D**) GE: germination energy; WD: wood distillate. Different letters indicate statistically significant differences at *p* < 0.05.

For *C. cyanus*, WD did not influence GP and GE at any of the tested concentrations. WD effects on MGT were variable according to the concentration, since no difference was found from 0% up to 1%, while there was a significant increase at a 2% concentration compared to 0.125% and 0.5% WD. GRI increased in dishes treated with 0.125% and 0.25% WD, while at higher concentrations there were no differences with respect to the controls (Figure 2).

**Figure 3:**
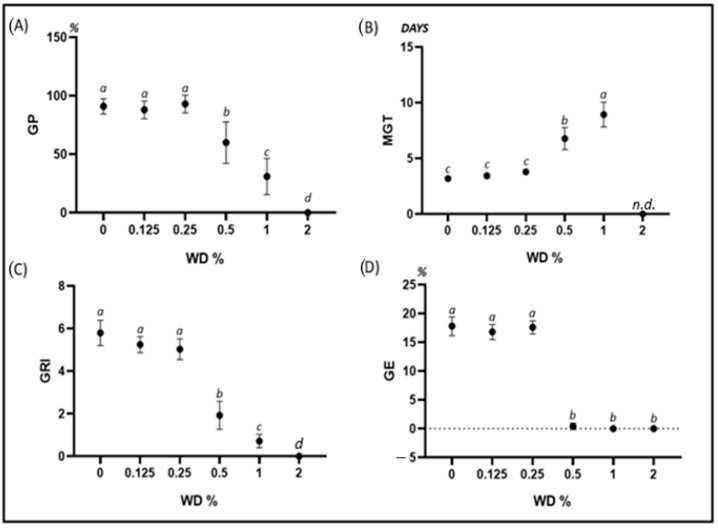
Error bar charts (mean ± standard deviation) showing the differences in the investigated germination parameters under different WD treatments on *Legousia speculum-veneris*. (**A**) GP: germination percentage; (**B**) MGT: mean germination time; (**C**) GRI: germination rate index; (**D**) GE: germination energy; WD: wood distillate. Different letters indicate statistically significant differences at *p* < 0.05. n.d.: not determined.

A different trend was observed on the germination parameters of L. speculum-veneris. In this species, WD clearly inhibited germination from 0.5% WD and at higher concentrations. Conversely, no effects were detected up to 0.25% WD for all the investigated parameters. Conversely, 0.5% WD or more caused a reduction in GP, GRI, and GE and an increase in MGT. At 2%WD, the germination was fully inhibited (Figure 3).

## 3. Discussion

Our work has highlighted how, at the low concentrations typically used to promote crop plant growth (0.2 to 0.5% WD [34,35,36]), WD has no negative effects on the germination performance of the three arable plant species investigated, with the exception of *Legousia speculum-veneris*, whose germination already began to be negatively affected at 0.5% WD. This is consistent with previous evidence showing that WD at such concentrations is not detrimental for *Bromus secalinus*, *Centaurea cyanus*, and *Legousia speculum-veneris* as regards their seedling emergence and first-stage development [24]. With regard to germination, there was contrasting evidence on the effects of WD depending on the plant species, but no negative effects were observed [27,37]. These findings are of significant interest as they highlight that WD has the potential to increase yield and improve the quality of crops without harming non-target plant species.

We observed that, with different effects according to the species, high concentrations of WD (0.5 to 2%) worsen the germination performance of arable plants, but a concentration lower than 0.5% WD (0.125 to 0.25%) never affects germination performance. Consistently, WD at high concentrations is an effective herbicide that is used for weed management in both agriculture and the conservation of cultural heritage, rather than for plant growth promotion [38,39,40]. It is unclear which of the components of WD exert such negative effects on seed germination. In fact, due to the complex chemical composition of WD, it is difficult to disentangle the effects of its single components [27]. However, acetic acid seems to be one of the most active compounds inhibiting plant development [41].

*L. speculum-veneris* was the species most sensitive to high WD concentrations, followed by *B. secalinus*. On the contrary, *C. cyanus* was the most tolerant species. This evidence is consistent with the biogeography and ecological specialization of these species, the latter being reflected in the width of their distribution range. *L. speculum-veneris* is restricted to Mediterranean areas, *B. secalinus* has a wider Eurosiberian distribution, and *C. cyanus* went cosmopolitan after its spread with crop seeds [42]. From this perspective, it is known that species with a wider distribution range are usually more competitive and stress-tolerant than species with a narrow distribution range, since they are adapted to a wider range of ecological conditions [43,44,45].

The differential response to the various treatments observed in the tested species may also be due to the diaspore size. Seeds of *B. secalinus* and achenes of *C. cyanus* have a mean size of 3 and 5 mm, respectively, while *L. speculum-veneris* seeds are about 1–2 mm long. It is known that seed size is correlated with the size of the outer layer, i.e., the pericarp or integument [46]. Consequently, bigger seeds have a thicker coat and a higher ability to withstand external stress [47]. The pericarp has an essential role in safeguarding the seed during dormancy and in ensuring its survival under unfavorable conditions [48]. The level of seed tolerance to a given substance also depends on its pericarp permeability. Impermeable or low-permeability pericarps may prevent or limit the absorption of substances by the seed. Conversely, permeable or moderately permeable pericarps facilitate the income of substances, promoting their uptake. It is worth noting that pericarp permeability can not only vary among different plant species, but also within the same species, being influenced by specific seed characteristics [49]. Additionally, seed size does not always correlate with relative germination speed [50]. It has been shown that species with small seeds only occasionally have additional adaptations for rapid germination, beyond the inherent advantage of small size for dispersal in the environment [50]. In our study, this tendency was not evident, as lower MGT was found for *C. cyanus* but not for *L. speculum-veneris*.

## 4. Materials and Methods

### 4.1. Selected Plant Species

**Figure 4:**
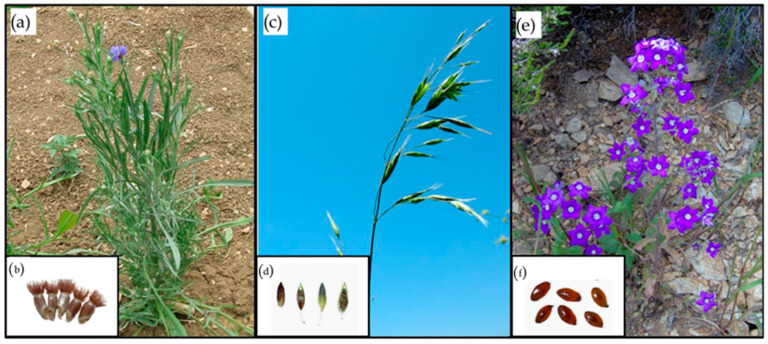
Studied species: *Centaurea cyanus* plant (**a**) and achenes (**b**); *Bromus secalinus* plant (**c**) and seeds (**d**); *Legousia speculum-veneris* plant (**e**) and seeds (**f**). Images retrieved from [53,54,55].

The investigated species were *Bromus secalinus* L. (Poaceae), *Centaurea cyanus* L. (Asteraceae), and *Legousia speculum-veneris* (L.) Chaix (Campanulaceae), which are all included in the list of the rarest/most threatened European arable plants (Figure 4) [7]. All the species are annuals and exhibit a winter–spring life cycle, being closely associated with winter cereal crops or related crop types [51]. *Bromus secalinus* is distributed across Europe and temperate Asia, especially at high latitudes. *Centaurea cyanus* originated in the Mediterranean area, but then went cosmopolitan after spreading with crop seeds and being grown as an ornamental plant. The range of *Legousia speculum-veneris* is currently restricted to the Mediterranean basin [43].

The seeds of the three species were requested through the “Botanic Gardens Conservation International” network [52]. The Agro-Botanical Garden of the University of Cluj-Napoca (Romania) provided the achenes of *C. cyanus*, while the seeds of *B. secalinus* and *L. speculum-veneris* were supplied by the Botanical Garden of the Ulm University (Germany).

### 4.2. Wood Distillate

Wood pyrolysis has two main residual components: a solid component known as biochar and a liquid component referred to as WD or pyroligneous acid [56]. To maximize the quantity of WD compared to that of biochar, the most effective production method is a fast pyrolysis caried out at temperatures ranging from 350 to 500 °C. This process entails a rapid heating of woody biomass, followed by a fast cooling. As a result, approximately 60–75% of the product comprises the liquid component, while the solid component makes up 15–25%, and the gaseous component contributes for 10–20% of the overall product. The composition of WD primarily consists of 80–90% water, along with over 200 water-soluble organic chemical compounds encompassing organic acids, alkanes, phenolics, alcohols, and esters [57,58,59]. The WD used in this experiment was produced via the pyrolysis of sweet chestnut (*Castanea sativa* Mill.) wood originating from forest management residuals. Its pH ranges from 3.5 to 4.5 and its density is of 1.05 kg/L. Its main components are 2–2.3% acetic acid, 2.9–3.02 g/kg of phenols, and 23–26 g/kg of polyphenols. The data from the producer indicate the following composition: total organic compounds = 33.8 g/L; organic acids = 32.3 g/kg; phenolic compounds = 13 g/L; methanol = 13.4 g/L; total N = 0.43 g/L [60]. Moreover, our still-unpublished data indicate: Ca = 326 mg/L; Na = 104 mg/L; K = 24 mg/L; P = 7 mg/L; Mg = 7 mg/L.

### 4.3. Seed Germination Assay

Seeds were surface sterilized via immersion in 3% sodium hypochlorite (NaClO) for two minutes and then washed thoroughly with distilled H_2_O. Subsequently, Petri dishes with a Whatman N1 filter paper (Whatman International, Maidstone, UK) were prepared and immediately soaked with treatment solutions. The WD concentrations tested were 0% (control), 0.125%, 0.25%, 0.5%, 1%, and 2%. For each species, 30 Petri dishes (5 for each treatment, statistical replicates) were prepared, and 20 seeds were laid in each dish. The Petri dishes were then placed in a growth chamber with a day/night cycle of 12 h/12 h and a 20 ± 3 °C temperature. From the time of sowing until the end of the experiment, the number of germinated seeds were recorded every day in order to calculate the germination percentage, the mean germination time, the Germination Rate Index, and the germination energy. The experiment was concluded when no more germination occurred after 7 days.

### 4.4. Germination Parameters

Germination performance was assessed through the following parameters:

I. Germination percentage (GP), calculated according to Czabator [61]:

$$GP\left(\%\right)=\left(\frac{Number\;of\;total\;germinated\;seeds}{Total\;number\;of\;tested\;seeds}\right)\times 100$$

II. Mean germination time (MGT), calculated according to Ellis and Roberts [62]:

MGT=∑(n×d)/N

where *n* is the number of seeds germinated on each day, *d* is the number of days from the beginning of the test, and *N* is the total number of seeds germinated at the end of the experiment;

III. Germination rate index (GRI), calculated according to Hossein et al. [63]:

GRI=∑ (Gn/Dn)

where *Gn* is the number of germinated seeds and *Dn* is the number of days since the beginning of observations.

IV. Germination energy (GE), calculated according to Czabator [61]:

$$GE\;\left(\%\right)=\left(\frac{Number\;of\;germinated\;seeds\;at\;4\;DAS}{Total\;number\;of\;tested\;seeds}\right)\times 100$$

where *DAS* is the number of days after sowing;

### 4.5. Statistical Analyses

Since the distribution of the values of some response variables was not normal, the values of MGT and GRI were square-root transformed and the values of GP and GE were log(x + 1) transformed to improve the normality of the data. The effect of WD addition on GP, MGT, GRI, and GE for the three selected species was assessed by means of one-way ANOVA tests, using the function, *aov*, in the package, *stats*, of R [64]. For significant results of the main test, we carried out post-hoc Tukey tests using the function *TukeyHSD* in the package, stats. We set α at 0.05. Graphs were created using the GraphPad Prism (version 8.4.1) software [65].

## 5. Conclusions

With this study, we have provided further evidence that the use of low-concentration (<0.5%) WD in agriculture is a sustainable practice, promoting higher yields and a higher quality of crop plants without harming arable plant diversity. This makes WD a very promising product for the ecologically sustainable management of crops and arable plant communities. Further investigation under open-field conditions will be useful to better understand the effects of WD on the germination parameters of arable plants under real cultivation conditions, where the effect of atmospheric agents and of other external factors may influence their response to the treatments.

## Figures and Tables

**Table 1:** Effects of different wood distillate addition on the germination parameters of the three selected species according to one-way ANOVA. GP: germination percentage; MGT: mean germination time; GRI: germination rate index; GE: germination energy. WD: wood distillate.

				*Bromus secalinus* L.					
Source of variation		**GP**		**MGT**		**GRI**		**GE**	
	*df*	MS	F	MS	F	MS	F	MS	F
WD addition	5	2.33	94.33 ***	1.53	26.73 ***	2.64	58.65 ***	0.92	125.4 ***
Residuals	24	0.02		0.06		0.05		0.01	
				*Centaurea cyanus* L.					
Source of variation		**GP**		**MGT**		**GRI**		**GE**	
	*df*	MS	F	MS	F	MS	F	MS	F
WD addition	5	0.03	2.34	0.11	3.81 *	0.34	4.47 **	0.02	2.20
Residuals	24	0.01		0.69		0.08		0.01	
				*Legousia speculum-veneris* (L.) Chaix					
Source of variation		**GP**		**MGT**		**GRI**		**GE**	
	*df*	MS	F	MS	F	MS	F	MS	F
WD addition	5	2.96	188.6 ***	5.29	405.1 ***	4.71	200.7 ***	2.26	447.2 ***
Residuals	24	0.01		0.01		0.02		0.01	

* = *p* < 0.05; ** = *p* < 0.01; *** = *p* < 0.001.

## Data Availability

Data are available on reasonable request by the corresponding authors.
